# Health-Related Quality of Life, Pain and Sleep in Patients with HIV Depending on the Clinical Situation: A Cross-Sectional Pilot Study

**DOI:** 10.3390/tropicalmed7120409

**Published:** 2022-11-30

**Authors:** Jon Salmanton-García, Francisco Alburquerque-Sendín, Javier Martín-Vallejo, Alicia Iglesias-Gómez, Miguel Cordero-Sánchez

**Affiliations:** 1 Faculty of Pharmacy, University of Salamanca, 37007 Salamanca, Spain; 2 University of Cologne, Faculty of Medicine, University Hospital Cologne, Translational Research, Cologne Excellence Cluster on Cellular Stress Responses in Aging-Associated Diseases (CECAD), 50931 Cologne, Germany; 3 University of Cologne, Faculty of Medicine, University Hospital Cologne, Department I of Internal Medicine, Center for Integrated Oncology Aachen Bonn Cologne Duesseldorf (CIO ABCD) and Excellence Center for Medical Mycology (ECMM), 50931 Cologne, Germany; 4 Department of Nursing, Pharmacology and Physical Therapy, Faculty of Medicine and Nursing, University of Córdoba, 14004 Córdoba, Spain; 5 Maimonides Biomedical Research Institute of Cordoba (IMIBIC), 14004 Córdoba, Spain; 6 Departamento de Estadística, Facultad de Medicina, Universidad de Salamanca, Campus Miguel de Unamuno, 37007 Salamanca, Spain; 7 Biostatistics Unit, Institute of Biomedical Research of Salamanca (IBSAL), 37007 Salamanca, Spain; 8 Servicio de Medicina Interna, Sección de Enfermedades Infecciosas, Complejo Asistencial Universitario de Salamanca (CAUSA), Instituto de Investigación Biomédica de Salamanca (IBSAL), Centro de Investigación de Enfermedades Tropicales de la Universidad de Salamanca (CIETUS), 37007 Salamanca, Spain

**Keywords:** HIV, quality of life, pain, sleep

## Abstract

Introduction: Health-related quality of life (HRQL), pain and sleep have been described as relevant traits in patients with human immunodeficiency virus (HIV). The aim of this study is to describe and evaluate HRQL, pain and sleep and their interdependence in HIV-positive patients. Methods: A cross-sectional study on HIV-infected patients was conducted. A set of five different questionnaires was used: two questionnaires addressing HRQL (Short Form 36 [SF-36] Health Survey and Medical Outcomes Study Short Form 30 [MOS-SF 30]), one on pain (McGill Pain Questionnaire) and one on sleep (Pittsburgh Sleep Quality Index [PSQI]). We also collected the sociodemographic and clinical characteristics of patients. Results: The sample included 109 patients (age: 46.08 ± 10.49 years; 68.8% male). The pain experience was independent of HRQL and sleep. Relationships among HRQL, pain and sleep associated to sociodemographic and clinical factors were not detected (*p* > 0.05). Patients with CDC category A showed moderate to high correlations among HRQL, pain and sleep. In CDC B-type patients, a moderate correlation was observed between pain and mental health. In CDC C patients, moderate correlations were observed between HRQL and sleep and pain and sleep, with no correlations between HRQL and pain. Conclusions: HRQL, pain and sleep are differently correlated in HIV patients depending on their clinical stage. Neither the nadir of CD4-T cells nor the current count of CD4-T cells was found to be related with HRQL, pain or sleep.

## 1. Introduction

In 2013, human immunodeficiency virus (HIV) had a prevalence of 34 million people infected throughout the world, with an estimated incidence of 2.5 million cases in 2012 [1]. In Spain, 2907 new cases were diagnosed during the same period with a mean ratio of 9/100,000 individuals [2]. After the appearance of antiretroviral therapy (ART) in 1996, HIV patients live longer and are not only interested in prolonging their life expectancy, but also to have a good health-related quality of life (HRQL) [3]. Several authors have shown that HIV infections correlate with a lower HRQL [3,4]. HRQL has been studied using different tools in HIV patients, including both generic [5] and HIV-specific [6] questionnaires.

Pain in patients with HIV has been shown to be extremely prevalent (between 40 and 60%), and the syndromes and causes of pain in out-patients with acquired immune deficiency syndrome (AIDS) have been documented too [7,8]. Furthermore, it was demonstrated that the HIV–pain relationship has high pharmaceutical costs, estimated at USD 13,276.81 per patient per year [7].

Sleep alterations are common in a high percentage of patients with HIV, ranging between 56%8 and 76% [9]. They constitute an important problem for the HIV population, closely related with an overall decrease in HRQL [9]. The diagnoses of AIDS, the CD4-T cell count, the ratio between CD4-T cells and CD8-T cells or the viral load do not affect sleep in seropositive people, but there is a relationship between the time living with the virus and receiving ART and sleep quality [10].

Nevertheless, the relationships among HRQL, pain, sleep and other clinical parameters are not well established. Accordingly, the present study aims to describe HRQL, pain and sleep and their relation with clinical and sociodemographic variables in HIV-positive patients in a follow-up scheme at a tertiary care service in Salamanca (Spain).

## 2. Methods

### 2.1. Samples and Procedures

A cross-sectional study with a non-probabilistic sampling of consecutive cases was designed. This was implemented in patients positive for HIV at the University Hospital in Salamanca (Spain), a reference hospital for 350,000 inhabitants, between November 2011 and January 2012.

Sample recruitment was performed through a proposal by the physician after completing a routine consultation. In case of acceptance, patients were informed as to the nature of this study, and informed written consent was obtained. A single trained observer collected the data individually from each patient. This study was approved by the Bioethics Committee of the University of Salamanca.

The criteria for inclusion in this study were as follows: the patient is diagnosed with HIV, the patient is receiving treatment at the consultancy of infectious diseases, the patient is aged above 18 and the patient signed a consent form to participate in this study. Exclusion criteria were less than 6 months from diagnosis, cognitive disorders involving comprehension and/or communication, and refusal to participate in this study.

### 2.2. Measures

The following sociodemographic and clinical variables were collected for this study: sex, age, weight, height, body mass index (BMI), educational level, marital status, domestic situation, income, employment status, substance abuse, treatment with methadone, months after diagnosis, HIV transmission practice, CDC category (expression of infection severity and progression), current CD4-T cell count, nadir of CD4-T cells, peak viral load, current viral load and opportunistic diseases.

HQRL was analyzed using the Spanish validated Short Form 36 Health Survey (SF-36). This is divided into 36 items grouped in 8 domains, namely: physical functioning, physical role, bodily pain and general health (grouped in the physical component summary (PCS) score), vitality, social functioning, emotional role and mental health (grouped in the mental component summary (MCS) score). The score ranges between 0 points for a low level of HQRL and 100, indicating a high HQRL level. It has a high internal consistency (Cronbach’s α = 0.7), and its test–retest reproducibility is between 0.58 and 0.99 [11].

We also applied the Medical Outcomes Study Short Form 30 (MOS-SF-30) specific for HIV. This is divided into 22 items and subdivided into 11 domains: general health perception, physical functioning, role functioning, social functioning, cognitive functioning, pain, mental health, energy/fatigue, health distress, quality of life and health transition. Scores ranged between 0 points for a low HRQL level and 100 points, indicating a high HQRL level. The questionnaire has a high consistency (Cronbach’s α = 0.78–0.89), and its test–retest reproducibility ranges between 0.58 and 0.85 [6].

The Spanish version of the McGill Pain Questionnaire (MPQ), divided into 64 descriptors organized into 19 items, was used to assess pain. Based on these items, the descriptors were hierarchized in order of intensity. The items were combined to achieve three domains: sensory (1–15), affective (16–18) and evaluative (19) as well as the sum of the three, called pain rating index (PRI). We also considered the number of words chosen by the patient (NWC) and the present pain index (PPI) with levels between 1 (slight pain) and 4 (pain was unbearable). The test showed a high consistency (Cronbach’s α = 0.84) [12].

Finally, we used the Pittsburgh Sleep Quality Index (PSQI) in its Spanish version. This is divided into 19 items that analyze the determinant factors of sleep quality grouped in 7 domains: sleep quality, sleep latency, sleep duration, habitual sleep efficiency, sleep disturbance, use of sleeping medication and daytime dysfunction. A higher score than 5 indicated poor sleep quality. The PSQI has shown a satisfactory reliability coefficient (0.78), and the component-total score correlations were all significant (0.53–0.77) [13].

### 2.3. Data analytic Strategy

For descriptive analyses, we calculated means and standard deviations of the quantitative variables. For qualitative variables, frequencies and percentages were used. To identify differences in the sociodemographic variables between patients, the statistical χ^2^, the Student’s *t* test and ANOVA tests were used, as appropriate, as well as the Kolmogorov–Smirnov test to identify the normal distribution of the quantitative data (*p* > 0.05).

To analyze the behavior with respect to the domains of the HRQL variables, pain, and sleep, we performed a biplot of principal components. The information is presented as a chart showing the variables and individuals on the same plane. To interpret the factors according to the analyzed domains, the corresponding vector angle should be used. Smaller angles of the domain with the factor indicate a larger weight of that variable in the factor considered. In addition, the angles between domains indicate associations among them. Sharp and flat angles represent strong associations (direct and indirect, respectively), and right angles indicate correlations close to zero. The number of factors chosen for the representation was made using the screen plot, selecting factors for which the greatest number of differences in the absorption of variance was observed. Ward’s cluster analysis was used to interpret the position of individuals on the biplot plane. The cluster was calculated by the coordinates of individuals from the biplot of principal components.

Owing to the high number of domains chosen and their associated interdependences, a multivariate analysis of variance (MANOVA) with Roy’s max root test was carried out to analyze the differences between the most relevant factors (sex, domestic situation, opportunistic disease, HIV transmission practice and substance abuse) with respect to all the HRQL domains, sleep and pain. We then calculated Pearson’s r coefficient as an estimator of the size of the effect to analyze the associations among domains.

Classical statistical analyses of the data acquired were performed using the JMP v.7 statistical package (SAS Institute Inc., Cary, NC, USA). The biplot of principal components and Ward’s cluster analysis were performed using MULTIBIPLOT (multivariate analysis using biplot) [14,15].

The level of significance was set to 0.05.

## 3. Results

A total sample of 121 consecutive patients was requested, of which 12 refused to join this study. Out of the 109 who accepted, 75 were men (68.8%). The mean age was 46.08 ± 10.49 years (range 21–79) with no significant differences between sexes (women = 46.2 ± 10.61 years/men = 46 ± 10.5 years; *p* = 0.957). The CD4-T cell count ranged between 40 and 1355 (469.16 ± 255.96 units). For the nadir of CD4-T cells, the range was between 2 and 600 (154.47 ± 119.74 units). In 70.6% of the cases, the current viral load was undetectable. Some individuals had not completed primary school (11.9%) or a university education (12.8%), whereas the most common level was primary school (55%). Regarding their domestic situation, there were significant differences between sexes (*p* = 0.01) since men lived with relatives 12 times more often than women; five lived with friends (none of them a woman), and twice the number of men stated that they lived alone. Moreover, for each single woman, there were four single men, whereas for each widower man, there were six women (*p* < 0.01).

The employment situation did not reveal differences between the unemployed, students, housewives, those working outside the home and pensioners. Monthly incomes differed as a function of sex (*p* = 0.014) with men earning higher salaries (94.11% of the subjects earning more than 1500 €/month were men).

Most patients in this study had a history of self-reported substance abuse (56%) without differences in sex. However, five times more men than women used methadone as substitutive therapy (*p* = 0.046). The sex distribution according to the HIV transmission practice was not statistically significant, except in the homosexual group which lacked women for comparison. A total of 43.11% of the population had been intravenous drug users (IDU) (21.27% women); 15.60% were homosexual men; 39.45% were individuals infected in heterosexual relations (55.81% women) and 18.3% had been infected in other circumstances (Table 1).

Regarding HRQL using SF-36, PCS was slightly higher than MCS. Here, the two domains most affected were general health and self-perception of quality of life. Physical functioning and physical role had the highest values, followed by social functioning and pain.

For MPQ, 40.4% of the patients referred no pain. This reflects the low mean values with high variability in the different rating indices: (PRI) (13.32 ± 13.54), NWC (6.44 ± 5.86) and PPI (1.67 ± 1.76), suggesting a heterogeneous presence of pain in these HIV patients.

Overall, values for sleep showed little variation, although the variability in the data was high, both regarding the total score (7.14 ± 4.44) and some components, such as sleep duration (0.95 ± 1.15), habitual sleep efficiency, (1.07 ± 1.27), use of sleeping medication (0.95 ± 1.35) and daytime dysfunction (0.64 ± 0.86). The least compromised component was daytime dysfunction, while sleep latency and sleep disturbance were the most affected. Nevertheless, the mode never surpassed a value of 1. Results of these questionnaires are shown in Table 2.

**Figure 1:**
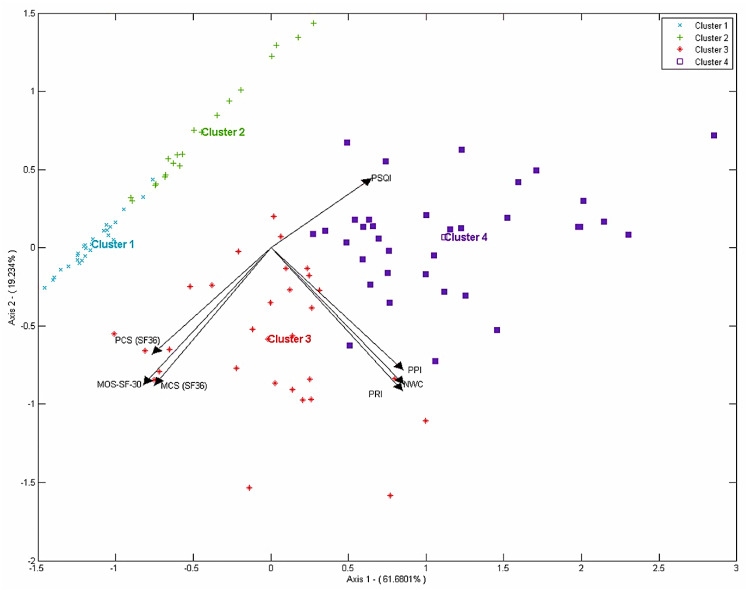
Representation biplot of the questionnaires applied per variable and patient. MCS, mental component summary score; MOS-SF-30, Medical Outcomes Study Short Form Survey 30; MPQ, McGill Pain Questionnaire; NWC, number of words chosen; PCS, physical component summary score; PPI, present pain intensity; PRI, pain rating index; PSQI, Pittsburgh Sleep Quality Index; SF-36, Short Form 36 Health Survey.

In the biplot of principal components, the first two factors, represented by the axes, accounted for 80.91% of the overall variance. The biplot representation shows the global dependence structure among the different domains related to HRQL, pain and sleep. It may be observed that the HRQL domains were inversely associated to the sleep domain and, to a lesser extent, to the pain domains. A weaker positive relationship was present between sleep and pain. Although all domains define the two factors, the first one is determined more by the HRQL and sleep domains, while the second factor of the analysis is determined more by the pain-associated domains. Ward’s cluster method from the individual coordinates was used to analyze the disposition of individuals. Based on the disposition of the individuals on the chart, two classifications can be made. The first one is defined by the pain domains, and it divides the individuals into two differentiated groups: those without pain (cluster 1&2)) and those with pain (cluster 3&4). The second classification is determined by the HRQL and sleep domains and is defined by a slope from the left to the right side of the plot with individuals with a high HRQL and sleep quality (clusters 1&3) and those with a low HRQL and sleep quality (clusters 2&4) (Figure 1).

The MANOVA Roy’s max root tests of the HRQL domains, sleep and pain between the groups defined by the sociodemographic and clinical factors did not find statistically significant differences dependent on the factors analyzed: sex: F = 0.976/*p* = 0.927; domestic situation: F = 0.783/*p* = 0.628, F = 0.984/*p* = 0.984; HIV transmission practice: F = 0.766/*p* = 0.155; and opportunistic diseases: F = 0.982/*p* = 0.967.

None of the CD4-T cell variables showed correlations with HRQL, pain or sleep (Table 3). A moderate inverse correlation was observed between HRQL, pain and sleep and being infected with HIV, although this pattern was modified as a function of the CDC category of the patients.

In CDC A patients, strong inverse correlations were observed between HRQL components and MPQ variables and between HRQL components and PSQI, and moderate to high direct correlations were observed between MPQ variables and PSQI. The nadir of CD4-T cells and the current CD4-T cell count were moderately directly correlated.

In CDC B patients, moderate inverse correlations were found only between MCS and MPQ variables and between MOS-SF-30 and PPI and PSQI. The nadir of CD4-T cells and the current CD4-T cell count were also directly correlated.

Finally, CDC C patients showed a consistent trend of correlations between PSQI and HRQL and MPQ variables. They were direct between pain and sleep and inverse between HRQL and sleep. There were no correlations between HRQL and pain, and neither between CD4-T-cell data (Table 3).

## 4. Discussion

The main aim of the present study is to describe and analyze the interrelationships of HRQL, pain and sleep with sociodemographic and clinical factors in a convenient sample of Spanish HIV patients. The observed changes in HIV infections in terms of the number of people affected, their sociodemographic characteristics as well as the distribution of infected individuals due to HIV transmission practices [16] determine the need to obtain a more precise description of the current characteristics of populations and samples. In this sense, the characteristics of our patients are similar to those reported in the Spanish National Registry of AIDS [17] and to those reported in previous Spanish [18,19] and international studies [8]. However, there seems to be discrepancies with studies reporting other specific populations (for example, the geographic location [5], the presence of lipodystrophy [20] and women with advanced stages of the disease) [21], which could affect the comparison of the results. The presence of self-reported substance abuse is a differential factor in our patients, with more men using the IDU transmission practice, reflecting the sex distribution in the general IDU population in Spain [22]. Regarding the clinical situation of the patients based on the CDC category, AIDS patients represented 32.1% of the sample, a figure similar to that reported in other studies [5,18,19,20]. These data suggest delayed diagnoses, a common phenomenon throughout Europe [23].

Results of the HRQL, pain and sleep in the overall sample, which account for 80.91% of the total variance, show that only sleep and HRQL are related in this case inversely (a higher score on PSQI and a lower one on HRQL), while pain is a fluctuating experience independent of HRQL and sleep. The relationships between HRQL and HIV [4,18] and between HRQL, sleep and HIV infection [24] have been described previously in different populations. None of the three variables are related either to age, the nadir of CD4-T cells, or the current CD4-T cell count. On the contrary, correlations between HRQL, pain and sleep mostly depended on the patient’s CDC category. For CDC A, HRQL, pain and sleep were highly correlated. In CBC B, the highest correlations were between pain and MCS, and for CDC C, between sleep and HRQL and pain.

The HRQL results were appreciably higher (more than 20 points for both the PCS and MCS) than those reported by López-Bastida et al. in Canary Islands patients (Spain) according to their immunological situation [5]. In that study, CDC A patients had mean scores of 50.07 ± 10.55 for PCS and 39.52 ± 14.67 for MCS, whereas scores of the CDC B individuals were 47.44 ± 10.30 and 40.99 ± 13.24, respectively, and the CDC C scores were 46.07 ± 10.74 and 35.63 ± 13.88 points. The differences in our data persisted for PCS and MCS in Brazilian patients, although the domains of physical role, emotional role and general health had higher scores in the study by Soárez et al. [25]. Australian patients [26], with the sole exception of general health and mental health domains, had scores similar to those we found in our patients.

Few studies used MOS-SF-30 despite its good clinimetric characteristics before the current analysis [6]. Our results are similar to those obtained in the validation of the questionnaire, regarding both the total scores [27] and the correlations with pain values [6]. In contrast, we did not find associations between clinical parameters (AIDS), the CD4 count and viral load with the domains of HRQL, although these correlations were reported in other Spanish populations [6,28].

The use of a generic and an HIV-specific HRQL questionnaire was useful, as previously documented [29], in detecting differences in the behavior between MCS and PCS through SF-36 (i.e., the relationship between pain and MCS domains in CDC B patients was not seen in the PCS) and allowed for the simultaneous determination the relationship between HRQL and the other variables using MOS-SF-30.

Our patients referred slight to moderate pain, while 40.4% of them referred no pain. These levels are in line with those described by Lee et al. [8]. This low rate of pain could be because our patients did not associate pain with HIV infection. Although the correlation between pain and HRQL was in general weak, it was present at higher levels between pain and HRQL in patients with CDC A and CDC C, but only for the MCS in B patients’ case. This interesting link between mental health and pain was described by Davis et al. [7], although these authors did not split their patients among HIV-clinical categories. Even though the different behaviors among CDC categories have not been previously shown, we hypothesize that the patients’ perception of their disease could explain these results. Specifically, CDC A and C patients have a greater perception of their physical changes caused by the disease and its treatment, which can also affect their mental state, linking both physical and mental health to the experienced pain. In contrast, the CDC B patients are more stable clinically, which may explain the lack of relationship between their physical state and pain. No relationship was found between pain and the CD4-T cell count, nor between pain and age, in agreement with Lee et al. [8]. However, other authors have reported a relationship between the level of pain and the CD4-T cell count, although only in women with advanced stages of the disease [21].

The prevalence of sleep alterations in our patients (66.1% with PSQI > 5) lies within the reported parameters [8,9]. From a clinical point of view, no relationship was detected between the current CD4-T cell count or their nadir and sleep, as reported by Lee et al. [8], although other authors are not in agreement with this [10]. This difference may be because our patients were generally in a poorer state of health than the military personnel analyzed by Nokes et al. [10]. The relationship between HRQL and sleep has been detected by other authors in adult populations [9] and also in adolescents [24]. Additionally, the results support the notion that there are certain patients, CDC B individuals, who do not relate sleep quality with the effects of HRQL.

The present study has methodological limitations. One of the main ones is the limited sample size used (109 patients). However, there are published surveys with a similar patient number that showed relevant evidence in this research area [19,30]. Future studies with a broader series and longitudinal designs are necessary to confirm the present results. Likewise, the external validity of this study was compromised by the fact that all patients were receiving ART. This selection bias is present because patients who fail to adhere to therapy (a poorer health state) also fail to attend regular check-ups, as well as the fact that slow-progressors are called fewer times for consultations. This study does not have a blinded assessor, although the type of questionnaire (self-reported) and the absence of intervention minimized the possible bias. Finally, there are no directives for the application of questionnaires in studies with HIV patients, which compromises any comparison between our own data and those of other authors.

## 5. Conclusions

The results of our study show that, although pain appears as an independent experience, the relationship between HRQL, pain and sleep depend on the developmental stage of the disease in HIV-infected individuals. The greater the values of HRQL and sleep quality, the lower the values of pain observed in patients with CDC A and C, while in CDC B patients, only mental health is directly related to pain. No relationship was observed between the nadir of CD4-T cells or the current CD4-T cell count and HRQL, pain or sleep. Accordingly, although longitudinal studies are needed, the CDC category (A, B or C) should be considered when analyzing the HRQL state, pain and sleep in patients with HIV.

## Figures and Tables

**Table 1:** Sociodemographic, epidemiological and clinical characteristics of the sample.

		χ^2^ Student’s *t*	*p* Value
**Sex** (n (%) male)	75 (68.8)		
**Age** (years) (mean *±* SD)	46.08 *±* 10.49	−0.122	0.904
**Weight** (mean *±* SD)	68.65 *±* 13.41	7.004	<0.001 *
**Height** (mean *±* SD)	1.69 *±* 0.1	8.888	<0.001 *
**BMI** (mean *±* SD)	23.99 *±* 3.66	2.080	0.040
**Educational level** (n (%))			
Incomplete primary school	13 (11.9)	2.082	0.556
Primary school	60 (55)		
High school	22 (20.2)		
University	14 (12.8)		
**Marital status** (n (%))			
Single	50 (45.9)	23.466	<0.001 *
Married	29 (26.6)		
Separated or divorced	15 (13.8)		
Common-law relationship	1 (0.9)		
Widowed	14 (12.8)		
**Domestic situation** (n (%))			
Living with partner/children/both	51 (46.8)	15.017	0.005 *
Living with relatives	26 (23.9)		
Living with friends	5 (4.6)		
Living alone	16 (14.7)		
Other	11 (10.1)		
**Income** (n (%))			
<€1500	92 (84.4)	6.012	0.014 *
≥€1500	17 (15.6)		
**Employment status** (n (%))			
Unemployed/housewife/student	33 (30.3)	2.416	0.299
Receiving income	40 (36.7)		
Pensioner/retired	36 (33)		
**Self-reported substance abuse** (n (%))	61 (56)	0.183	0.669
**Treatment with methadone** (n (%))	26 (23.9)	3.975	0.046 *
**Months after diagnosis** (mean *±* SD)	191 *±* 94	<0.001	1.000
**HIV transmission practice** (n (%) male)			
IDU	37 (33.94)	22.705	<0.001 *
Heterosexual relationship (non IDU)	19 (17.43)		
Homosexual relationship (non IDU)	17 (15.6)		
Other	2 (1.8)		
**CDC category** (n (%))			
A	19 (14.72)	3.451	0.178
B	55 (50.45)		
C	35 (32.11)		
**Current CD4-T cell count** (n (%))			
<200	16 (13.6)	1.013	0.603
200–499	46 (42.2)		
≥500	47 (43.1)		
**Nadir of CD4-T cells** (n (%))			
<200	77 (70.6)	2.308	0.315
200–499	30 (27.5)		
≥500	2 (1.8)		
**Current viral load** (n (%))			
<40	74 (62.7)	0.799	0.850
40–1000	30 (25.4)		
>1000	5 (4.2)		
**Peak viral load** (n (%))			
<40	6 (5.5)	1.457	0.483
40–1000	21 (19.3)		
>1000	82 (75.2)		
**Opportunistic diseases** (n (%))			
None infection	17 (15.6)	1.470	0.689
B	58 (53.2)		
C	6 (5.5)		
B–C	28 (25.7)		

SD, standard deviation; BMI, body mass index; IDU, intravenous drug use; CDC, Centers for Disease Control and Prevention. * Indicates statistically significant differences between groups by sex.

**Table 2:** Mean scores and percentiles for the various SF-36, MOS-SF-30, MPQ and PSQI questionnaire domains.

	CDC A		CDC B		CDC C		Fisher’s F	*p* Value	OVERALL	
	Mean	SD	Mean	SD	Mean	SD			Mean	SD
**SF-36**										
*Physical Functioning*	85.26	25.63	85.46	21.68	78.14	31.67	0.950	0.390	83.12	25.93
*Physical Role*	71.05	44.30	80.00	36.77	67.86	43.12	1.061	0.350	74.54	40.25
*Bodily Pain*	71.05	34.88	79.86	27.36	59.93	36.69	1.084	0.342	71.93	32.86
*General health*	59.74	23.20	53.55	21.85	49.71	25.49	4.176	0.018 *	53.35	23.32
PCS	71.10	28.49	74.74	19.25	63.91	27.88	2.197	0.116	70.73	24.26
*Vitality*	68.68	28.18	65.36	22.63	59.00	29.70	1.060	0.350	64.04	26.07
*Social functioning*	75.66	28.40	73.18	29.89	71.07	31.03	0.147	0.863	72.94	29.78
*Emotional role*	70.17	41.42	75.15	38.61	59.05	44.35	1.657	0.196	69.11	41.26
*Mental health*	60.53	24.03	64.66	19.83	65.49	26.68	0.026	0.974	65.14	22.89
MCS	70.10	25.95	69.66	19.49	63.65	28.58	0.783	0.460	67.81	23.81
**MOS-SF-30**										
*General health perception*	2.00	1.05	1.95	0.90	1.49	1.04	2.600	0.079	1.81	1.03
*Physical functioning*	10.26	3.02	10.04	2.72	8.97	3.88	1.517	0.224	9.73	3.2
*Role functioning*	3.47	1.12	3.33	1.33	2.80	1.76	1.862	0.160	3.18	1.47
*Social functioning*	3.11	1.29	3.22	1.26	2.71	1.69	1.383	0.255	3.04	1.42
*Cognitive functioning*	12.11	3.70	11.42	4.05	10.97	3.86	0.514	0.600	11.39	3.92
*Pain*	2.58	1.26	2.76	1.29	2.51	1.31	0.434	0.649	2.65	1.29
*Mental health*	14.26	4.83	13.13	5.04	12.51	5.71	0.688	0.505	13.13	5.22
*Energy/fatigue*	10.68	4.85	10.55	3.97	10.03	5.19	0.182	0.834	10.40	4.51
*Health distress*	10.21	5.18	11.85	4.10	12.29	4.14	1.490	0.230	11.71	4.33
*Quality of life*	2.68	0.82	2.65	0.97	2.43	1.24	0.603	0.549	2.59	1.04
*Health transition*	2.37	1.01	2.58	0.81	2.26	0.89	1.563	0.214	2.44	0.88
Total score	73.74	19.65	73.47	16.42	68.97	21.20	0.716	0.491	72.07	18.58
**MPQ**										
*Sensory PRI*	8.74	8.63	6.13	6.67	11.20	9.39	4.378	0.015 *	8.09	8.24
*Affective PRI*	1.95	2.82	1.09	1.69	2.40	2.91	3.487	0.034 *	1.66	2.41
*Evaluative PRI*	1.37	1.50	1.18	1.38	1.83	1.45	2.237	0.112	1.42	1.44
*Miscellaneous PRI*	1.68	2.71	2.00	3.03	2.83	3.10	1.169	0.315	2.21	3.01
PRI	13.74	14.15	10.40	11.29	18.26	15.13	3.834	0.025 *	13.32	13.54
NWC	6.53	6.00	5.24	5.55	8.29	5.93	3.009	0.054	6.44	5.86
PPI	1.58	1.81	1.35	1.54	2.23	1.96	2.819	0.064	1.67	1.76
**PSQI**										
*Sleep quality*	0.95	0.97	0.96	0.61	1.11	0.96	0.444	0.643	1.01	0.80
*Sleep latency*	1.42	1.47	1.15	1.27	1.40	1.31	0.541	0.584	1.28	1.31
*Sleep duration*	0.84	1.12	0.84	1.10	1.20	1.23	1.183	0.310	0.95	1.15
*Habitual sleep efficiency*	1.00	1.25	0.98	1.28	1.26	1.29	0.533	0.589	1.07	1.27
*Sleep disturbance*	1.42	0.77	1.15	0.45	1.26	0.61	1.726	0.183	1.23	0.57
*Use of sleeping medication*	0.89	1.29	0.89	1.34	1.09	1.42	0.242	0.786	0.95	1.35
*Daytime dysfunction*	0.47	0.61	0.55	0.81	0.89	0.99	2.185	0.118	0.71	1.05
Total score	7.00	5.03	6.51	6.93	8.20	4.80	1.576	0.212	7.14	4.44

MCS, mental component summary score; MOS-SF-30, Medical Outcomes Study Short Form Survey 30; MPQ, McGill Pain Questionnaire; NWC, number of words chosen; PCS, physical component summary score; PPI, present pain intensity; PRI, pain rating index; PSQI, Pittsburgh Sleep Quality Index; SD, standard deviation; SF-36, Short Form 36 Health Survey. * Indicates statistically significant differences between groups.

**Table 3:** Correlations between quantitative variables of this study.

	CDC	Current CD4	PCS (SF-36)	MCS (SF-36)	MOS-SF-30	PRI (MPQ)	NWC (MPQ)	PPI (MPQ)	PSQI
**Nadir CD4**	Overall (n = 109)	0.475 *	0.032	−0.102	0.000	−0.059	−0.042	−0.023	−0.094
	A (n = 19)	0.537 *	−0.123	−0.247	−0.087	0.269	0.251	0.264	−0.083
	B (n = 55)	0.472 *	−0.058	−0.264	−0.102	−0.021	−0.013	0.063	0.019
	C (n = 35)	0.332	0.118	0.087	0.109	−0.158	−0.124	−0.170	−0.164
**Current CD4**	Overall (n = 109)		0.099	0.073	0.131	−0.088	−0.059	−0.081	−0.076
	A (n = 19)		−0.106	−0.172	0.018	−0.006	−0.031	−0.167	0.041
	B (n = 55)		−0.004	−0.057	0.028	−0.058	−0.017	0.012	−0.088
	C (n = 35)		0.283	0.297	0.283	−0.113	−0.078	−0.096	−0.077
**PCS (SF-36)**	Overall (n = 109)			0.620 *	0.760 *	−0.465 *	−0.468 *	−0.442 *	−0.462 *
	A (n = 19)			0.786 *	0.872 *	−0.891 *	−0.866 *	−0.710 *	−0.672 *
	B (n = 55)			0.517 *	0.658 *	−0.285	−0.291	−0.305	−0.096
	C (n = 35)			0.604 *	0.791 *	−0.334	−0.378	−0.369	−0.629 *
**MCS (SF-36)**	Overall (n = 109)				0.825 *	−0.386	−0.404 *	−0.439 *	−0.483 *
	A (n = 19)				0.832 *	−0.765 *	−0.713 *	−0.664 *	−0.694 *
	B (n = 55)				0.753 *	−0.479 *	−0.509 *	−0.572 *	−0.342
	C (n = 35)				0.879 *	−0.110	−0.126	−0.201	−0.484 *
**MOS-SF-30**	Overall (n = 109)					−0.438 *	−0.446 *	−0.496 *	−0.571 *
	A (n = 19)					−0.835 *	−0.809 *	−0.699 *	−0.664 *
	B (n = 55)					−0.356	−0.379	−0.481 *	−0.436 *
	C (n = 35)					−0.307	−0.320	−0.395	−0.642 *
**PRI (MPQ)**	Overall (n = 109)						0.948 *	0.875 *	0.410 *
	A (n = 19)						0.979 *	0.868 *	0.663 *
	B (n = 55)						0.945 *	0.864 *	0.224
	C (n = 35)						0.942 *	0.873 *	0.397 *
**NWC (MPQ)**	Overall (n = 109)							0.870 *	0.439 *
	A (n = 19)							0.837 *	0.632 *
	B (n = 55)							0.876 *	0.316
	C (n = 35)							0.869 *	0.422 *
**PPI (MPQ)**	Overall (n = 109)								0.415 *
	A (n = 19)								0.465 *
	B (n = 55)								0.279
	C (n = 35)								0.472 *

CDC, Centers for Disease Control and Prevention; SF-36, Short Form 36 Health Survey; PCS, physical component summary score; MCS, mental component summary Score; MOS-SF-30, Medical Outcomes Study Short Form 30; MPQ, McGill Pain Questionnaire; PRI, pain rating index; NWC, number of words chosen; PPI, present pain index; PSQI, Pittsburgh Sleep Quality Index. * Indicates statistically significant differences between groups.

## Data Availability

Data will be available upon reasonable request to the corresponding author.
